# A double-edged sword of immuno-microenvironment in cardiac homeostasis and injury repair

**DOI:** 10.1038/s41392-020-00455-6

**Published:** 2021-02-22

**Authors:** Kang Sun, Yi-yuan Li, Jin Jin

**Affiliations:** 1 MOE Laboratory of Biosystem Homeostasis and Protection, and Life Sciences Institute, Zhejiang University, Hangzhou, 310058 China; 2 Key Laboratory for Developmental Genes and Human Disease, Ministry of Education, Institute of Life Sciences, Jiangsu Province High-Tech Key Laboratory for Bio-Medical Research, Southeast University, Nanjing, 210096 China; 3 Sir Run Run Shaw Hospital, College of Medicine Zhejiang University, Hangzhou, 310016 China

**Keywords:** Adaptive immunity, Innate immunity, Cell biology

## Abstract

The response of immune cells in cardiac injury is divided into three continuous phases: inflammation, proliferation and maturation. The kinetics of the inflammatory and proliferation phases directly influence the tissue repair. In cardiac homeostasis, cardiac tissue resident macrophages (cTMs) phagocytose bacteria and apoptotic cells. Meanwhile, NK cells prevent the maturation and transport of inflammatory cells. After cardiac injury, cTMs phagocytose the dead cardiomyocytes (CMs), regulate the proliferation and angiogenesis of cardiac progenitor cells. NK cells prevent the cardiac fibrosis, and promote vascularization and angiogenesis. Type 1 macrophages trigger the cardioprotective responses and promote tissue fibrosis in the early stage. Reversely, type 2 macrophages promote cardiac remodeling and angiogenesis in the late stage. Circulating macrophages and neutrophils firstly lead to chronic inflammation by secreting proinflammatory cytokines, and then release anti-inflammatory cytokines and growth factors, which regulate cardiac remodeling. In this process, dendritic cells (DCs) mediate the regulation of monocyte and macrophage recruitment. Recruited eosinophils and Mast cells (MCs) release some mediators which contribute to coronary vasoconstriction, leukocyte recruitment, formation of new blood vessels, scar formation. In adaptive immunity, effector T cells, especially Th17 cells, lead to the pathogenesis of cardiac fibrosis, including the distal fibrosis and scar formation. CMs protectors, Treg cells, inhibit reduce the inflammatory response, then directly trigger the regeneration of local progenitor cell via IL-10. B cells reduce myocardial injury by preserving cardiac function during the resolution of inflammation.

## Introduction

In humans and animals, the heart is an indispensable organ for the survival. The cardiovascular system serves as the center of blood circulation, it pumps blood continuously by beating regularly to ensure energy supplies and material exchange in the body^1^. Cardiac injury is the current second major cause of death in modern trauma except brain trauma^2^. ischemic heart disease and stroke caused 12.9 million deaths in 2010, accounting for a quarter of the global deaths^3^, suggested by a statistics on the cause of death in 187 countries from 1980 to 2010. Therefore, it is important to clarify the detailed mechanism of the cardiac repair after injury for public health. The immune cells not only protect the host against invading pathogens, but also play vital roles in the repair and regeneration of damaged tissues^4–7^. Some researches on heart development reveal essential roles of immune cells in promoting cardiac homeostasis and injury repair, but these repair processes also increase “bystander damage” that overreacts to injury^8^.

## Cardiac tissue injury and repair

### Types of cardiac tissue injury

**Fig. 1:**
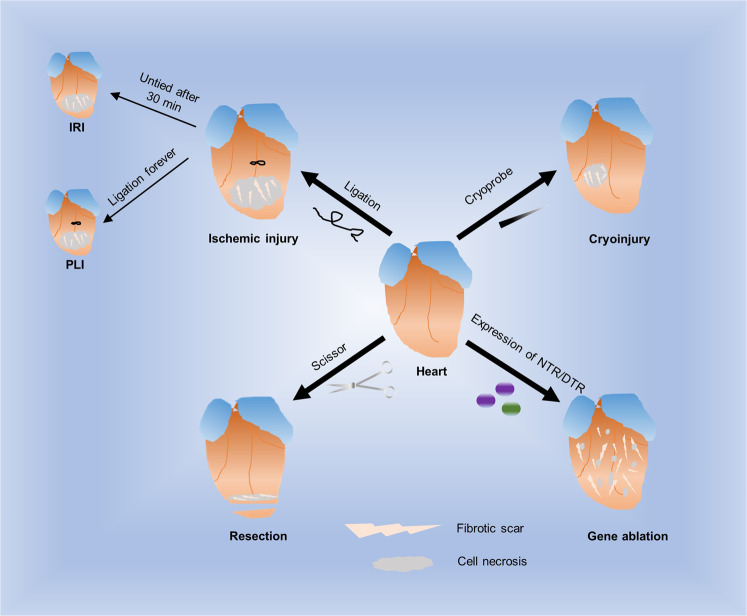
Types of cardiac tissue injury. Ischemia-reperfusion injury (IRI), permanent ligation injury (PLI), cryoinjury, resection, and gene ablation are the four main ways of studying cardiac injury and repair. IRI is used to simulate the pathological state of myocardial infarction (MI). The ligation is untied and blood reperfusion is performed after ligation of the mouse artery for 30 min Cryoinjury is caused by cauterization of ventricular tissue with a cryoprobe. Resection is used to surgically remove part of the cardiac tissues. The gene ablation method specifically expresses bacterial nitroreductase (NTR) or diphtheria toxin receptor (DTR) in cardiomyocytes (CMs) to result in CM death

The types of researched cardiac tissue injury usually include ischemic injury, cryoinjury, resection, and gene ablation. Ischemic injury mainly includes ischemia-reperfusion injury (IRI) and permanent ligation injury (PLI)^9,10^. At present, the mouse heart IRI model is often used to study the pathological state of myocardial infarction (MI)^11^. MI is an ischemic cardiac disease. Ischemic cardiac disease is the main cause of human death worldwide. The first symptom of patients with ischemic cardiac disease is acute MI, and then myocarditis occurs because of infarction^12–14^. Myocarditis further leads to ventricular dysfunction and eventually causes heart failure (HF)^15^. IRI leads to the loss of myocardial cells, and during the healing process, the injured myocardial tissues are gradually replaced by fibrotic scar tissues^16^. IRI is commonly induced by ligation of left anterior descending coronary arteries to cause ischemic death of cardiomyocytes in downstream tissues. The ligation is then untied, and blood reperfusion is performed after ligation of the mouse artery for 30 min^17^, but PLI is induced by ligating coronary artery forever. Cryoinjury is usually performed by cauterization of ventricular tissue with a metal probe or a cryoprobe balanced with liquid nitrogen^18^, and it is administered for different times according to the experimental requirements to control the degree of injury. This is also a very common method in the treatment of cardiac injury^19^. Cryoinjury also leads to obvious cell necrosis at the injury site and the formation of fibrotic scars. This type of injury model is also similar to the normal cardiac pathological state in humans^20–22^. In the laboratory, a mouse resection model that is used to simulate surgical removal of part of the cardiac tissue or a small part of the ventricle is suitable for almost all animals, but although the resection method can effectively cause tissue loss, compared with MI, this method only causes a small amount of damage to the injury site and surrounding tissue by cell necrosis and fibrotic scar formation^23,24^. Gene ablation is a type of cardiac injury that causes cardiomyocyte death without surgical operation. The current method for gene ablation is to specifically express nitroreductase (NTR) from bacteria or diphtheria toxin receptor (DTR) on cardiomyocytes (CMs)^25,26^. NTR induces cytotoxic products such as metronidazole (Mtz), and NTR expression induces CMs to become sensitive to diphtheria, which leads to CM death^27^. However, the problem with gene ablation methods is that the fibrotic scars produced are difficult to quantify and compare (Fig. 1)^6^.

### Repair of injured cardiac tissues

MI is the main ischemic cardiac disease after cardiac ischemic injury, which is defined as acute death of cardiac tissues^28^. There is a major unmet clinical need to treat cardiac ischemic injury, though preconditioning, postconditioning and remote conditioning of ischemic conditioning protect the heart from infarction with a lot of preclinical evidence, current phase II trials based on ischemic conditioning have different results for the treatment of cardiac ischemic injury^29^. Much effort aimed to temper the initial inflammatory response to preclinical models^30^ and patients has been done, because inflammatory response is vital to the repair process, but the clinical results are disappointing^31^. Most researches on cardiac ischemic injury and repair only focus on cytokines like interleukin-1β (IL1β) and tumor necrosis factors, antibodies to adhesion molecules of leukocyte invasion and the complement cascade^30,32^. Coronary ligation with 60 minutes can cause death of most cardiomyocytes in the subendocardial area^33^. Cardiomyocyte death cannot be confirmed for several hours after coronary ischemia, but irreversible changes have been induced in some cardiomyocytes of the subendocardial area after a 20–30 min interval of severe ischemia^34^. Current methods of repairing cardiac ischemic injury are divided into two categories: molecular therapy and cell therapy.

#### Molecular therapy

##### FGF2 reduces infarct size, and VEGFA increases angiogenesis

Growth factors are signaling molecules that contribute to various cellular processes. There are currently two growth factors that significantly improve cardiac function after MI: fibroblast growth factor 2 (FGF2) and the angiogenic factor vascular endothelial growth factor A (VEGFA). In contrast to wild-type mice, experimental mice with upregulated FGF2 after IRI exhibit reduced infarct sized and improved cardiac function. In addition, FGF2 deficiency exacerbates cardiac dysfunction after IRI^35^. In animal models of MI, the administration of VEGFA improves local coronary blood flow and restores cardiac function^36^. In addition, gene-based modification therapy, such as synthetically modified RNAs (modRNAs), modRNAs are nucleotides that one or more nucleotides replaced by modified nucleotides. In previous study, modRNAs have advantage of highly efficient expression of transient protein in vitro and in vivo, but they don’t elicit an innate immune response^37–42^. modRNAs increase the delivery and expression efficiency of VEGFA. First, in vitro experiments verified that VEGFA encoded by modRNAs has the new function of controlling the cell fate of pluripotent islet 1 + (Isl1 + ) human cardiac progenitor cells. VEGFA made these cells drive away from the fate of becoming cardiomyocytes but moved towards the fate of vascular endothelial cells^43,44^. Human VEGFA has been expressed by modRNAs in mouse hearts after MI. Compared with the application of DNA vectors^45–47^, modRNAs mediate the “pulse-like” expression of VEGFA in vivo, which has advantages in reducing infarct size, enhancing the function of myocardial perfusion and promoting survival^48^. To some extent, this effect is due to a new effect of VEGFA on epicardial progenitor cells: modRNA-mediated expression of VEGFA makes these progenitor cells proliferate, promotes their migration to cardiomyocytes, and redirects them to differentiate into vascular lineages^49^. These results indicate that modRNA-encoded VEGFA drives changes in the fate of cardiac progenitor cells in vivo^50–52^, thereby enhancing cardiac repair. In addition, the growth factor neuroregulatory protein 1 (NRG1) and its receptors, the tyrosine protein kinases ERBB2 and ERBB4, play key roles in formation of trabeculae and endocardial pads during cardiac development^53^. Activation of the NRG1-ERBB2/ERBB4 signaling pathway in the hearts of injured adult mice can induce cardiomyocyte proliferation and improve cardiac function^54,55^. Paired-like expression of homeodomain transcription factor 2 (Pitx2) in the hearts of newborn mice repairs cardiac injury after apical resection, while adult mouse cardiomyocytes expressing the Pitx2 gene effectively regenerate after myocardial infarction by regulating electron transport and scavenging reactive oxygen species (ROS)^56^. In addition, cardiac health and diseases are associated to the transcription factor NF-E2 related factor 2 (Nrf2). Multiple lines of evidence support the potential cardioprotective role of Nrf2. Nrf2 exerts a protective effect by reducing oxidative stress, apoptosis and inflammation^57,58^.

##### miRNAs mainly regulate cardiomyocyte proliferation

MiRNAs are a kind of noncoding small RNAs that are highly conserved, single-stranded RNAs. The coding gene is located in the noncoding region or the coding region containing the exons and introns. Mature miRNAs are approximately 22 bases in length. As new gene regulatory elements, miRNAs inhibit target gene transcription to regulate gene expression by binding to complementary sequences in the 3’-untranslated regions (UTRs) of mRNAs^59^. Some miRNAs activate DNA replication in CMs, and by using a library of 875 human miRNAs, a unbiased screening has detected a variety of miRNAs which induce the proliferation of neonatal rat cardiomyocytes^60^. In animal studies, it has been reported that other miRNAs, such as the miR-214, miR-302-367, miR-17-92 cluster, and the miR-222 cluster, also contribute to cardiac repair by inducing CM proliferation in vivo^61–64^. For example, overexpression of miR-199a or miR-590 induced the proliferation of adult mouse CMs in mouse hearts, and improved the cardiac function of adult mouse infarcted hearts, and reduced fibrosis. Therefore, the positive results of preclinical research prove the therapeutic potential of miRNAs.

##### Exosomes mediate communication between cardiomyocytes

**Fig. 2:**
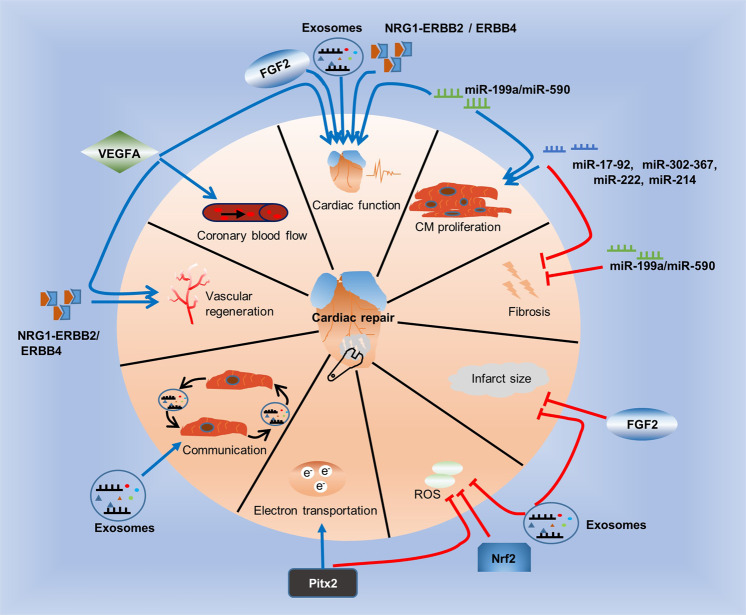
Molecular therapy of injured cardiac tissues. Some molecules promote repair (blue line), while others inhibit repair (red line) after cardiac injury. Growth factors such as VEGFA, FGF2, and NRG1, the NRG1 receptors ERBB2 and ERBB4, exosomes, and miR-199a or miR-590 restore cardiac function. miR-199a or miR-590, the miR-17-92 cluster, miR-214, miR-302-367, and miR-222 induce CM proliferation. The miR-17-92 cluster, miR-214, miR-302-367, miR-222 and miR-199a or miR-590 reduce fibrosis. FGF2 and exosomes reduce infarct size. Exosomes, Nrf2 and Pitx2 scavenge ROS. Pitx2 also regulates electron transport. Exosomes can act as mediators of cell-to-cell communication. NRG1 and its receptors ERBB2 and ERBB4 and VEGFA improve vascular regeneration. VEGFA also improves local coronary blood flow

Exosomes are a sort of small extracellular vesicles, their size is mainly 30–100 nm in diameter, which are produced by cells, they are characterized by some specific surface markers, such as CD9, CD63, and CD81^65^. Exosomes can act as mediators of communication between cells by carrying cell-specific mRNAs or miRNAs, and increasing research evidence supports the role of exosomes in cell communication between heart cells^66^. Today, small extracellular vesicles called exosomes are known as the key mediators of beneficial mesenchymal stem cell (MSC) paracrine effects^66,67^. Some miRNAs contained by exosomes have therapeutic significance by changing gene expression of recipient cells^68^. For example, miR-21a-5p is the main paracrine factor produced by MSCs, and plays a cardioprotective effect through the synergistic activity of multiple pathways^69^. The secretion of MSC-derived exosomes to the heart can reduce oxidative stress and promote the survival of cardiomyocytes after IRI in mice, further reducing infarct size and ameliorating cardiac function (Fig. 2)^70^.

#### Cell therapy

**Fig. 3:**
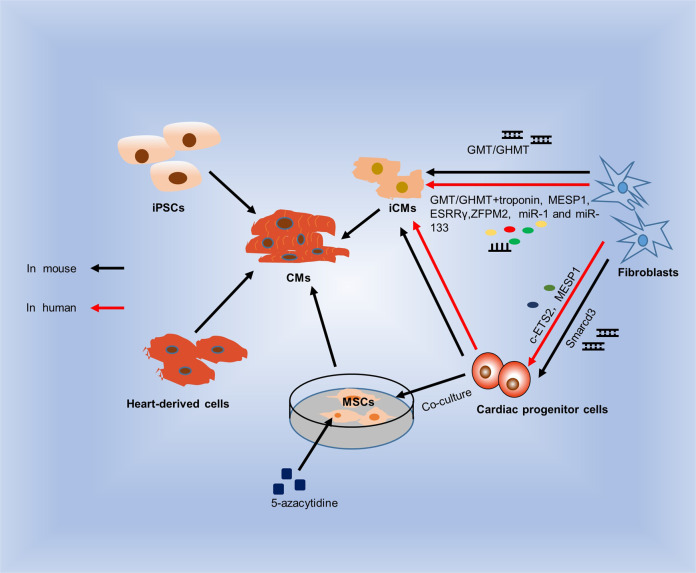
Cell therapy of injured cardiac tissues. iPSCs can differentiate into functional cardiomyocytes in vitro. Heart-derived cells can differentiate into many types of heart cells. GMT or GHMT mixtures reprogram mouse fibroblasts to differentiate into iCMs, and Smarcd3 reprograms mouse fibroblasts to differentiate into cardiac progenitor cells in vitro. MSCs differentiate into CMs in the presence of 5-aza or in coculture with cardiac progenitor cells. However, reprogramming human fibroblasts requires GMT or GHMT mixtures and troponin, MESP1, ESRRγ and ZFPM2, was well as miR-1 and miR-133. Human fibroblasts can be transformed into cardiac progenitor cells with c-ETS2 and MESP1

Currently, cell therapy usually involves the use of resident cardiomyocytes with stem-like characteristics for treatment. Preclinical studies used human embryonic stem cells (hESCs)^71^, and induced pluripotent stem cells (iPSCs)^72^, both are human pluripotent stem cells (hPSCs), they can differentiate into functional CMs in vitro^73^. In Clinical therapies, per patient dose may need 10^8^ to 10^9^ cells of hPSC-derived CMs, In addition, a large number of hPSC-derived CMs are needed to determine the safety or efficacy signals^74^. However, the current experimental data have always shown that hPSC-CM-mediated cardiac repair still has great challenges to clinical application, and the clinical goal seems difficult to achieve^75^. Heart-derived cells can expand, show pluripotency and differentiate into many heart-type cells in vitro^76^. Another method of repairing cardiac injury involves generating functional cardiomyocytes in vitro and then transplanting these cells into the injured heart. Before, to protect the failing heart, scientists initially adopted the first generation of cell-based therapies. Since sufficient cardiomyocytes were not available, first-generation cell therapy included the transfer of noncardiomyocytes. The initial candidate cells included skeletal muscle cells that were expected to promote cardiac contraction, as well as bone marrow-derived cells and MSCs that showed cardiogenic potential in vitro^77,78^. These cells have become the main source of cell-based therapy for HF. MSCs can differentiate into CMs by addition of DNA methyltransferase inhibitor 5-azacytidine (5-aza) or coculture with cardiac progenitor cells^79,80^. Though stem cell transplantation can form new blood vessels in animal models of MI, the clinical efficacy of stem cell transplantation in patients with MI is still unclear. Because of MSCs’ pluripotency, it may be dangerous if transplanting MSCs into human body^73^. “Paracrine hypothesis” is associated with stem cell-mediated cardiac repair, which is defined that stem cells release substances to improve injured and diseased myocardium and promote heart regeneration^81^. There are lots of evidence support that factors released by the autocrine and paracrine mechanisms from resident cardiac cells, could play an important role in repair process of the failing heart. Besides exosomes mentioned above, there are many other factors, which are released by the unconventional and the conventional secretory pathways^82^, also are in support of this hypothesis. Such as anti-apoptotic, pro-survival and angiogenic factors secreted by stem cells under particular condition^83^. It is found that nutritional factors by injecting a stem cells culture medium effectively promote heart repair in a mouse model of HF^84^. In the heart, the most studied paracrine factor is atrial natriuretic peptide because the ventricular myocytes in the diseased adult heart secrete atrial natriuretic peptides through constitutive conventional pathway in response to stretch and adrenaline stimulation, and healthy ventricular myocytes do not have the function of secreting granules^85^. In fact, if paracrine factors that improve heart function can be identified, pharmacological treatments based on these factors may be easier to translate into clinical applications than cell therapy^86^. Fibroblasts can be directly or partly reprogrammed to differentiate into cardiomyocyte-like cells or cardiac progenitor cells by overexpressing multiple transcription factors associated with cardiac development. One strategy for reprogramming fibroblasts into cardiomyocytes is to force the expression of five genes related to early heart factors, Mespl, Gata4, Tbx5, Nkx2-5, and Baf60c (also known as Smarcd3), to reprogram mouse fibroblasts into a scalable multipotent cardiac progenitor cell population, thus bypassing the multipotent state^87^. Induced expression of three genes related to cardiac transcription factors, Gata4, Mef2c and Tbx5, also called GMT, or a combination of GMT plus Hand2 (called GHMT) successfully reprogrammed mouse fibroblasts to differentiate into induced cardiomyocyte-like cells (iCMs) in vitro. iCMs express major cardiac genes and have cardiomyocyte characteristics, such as sarcomere structures, pulsatile contractions and spontaneous intracellular calcium oscillations, without undergoing cardiac precursor states^88^. However, GMT or GHMT reprogramming mixtures used for direct reprogramming of human fibroblasts require other factors, such as troponin, MESP1, estrogen-related receptor-γ (ESRRγ) and zinc finger protein ZFPM2, as well as some miRNAs such as miR-1 and miR-133, which are necessary to induce human fibroblasts to converse into cardiomyocyte-like cells^89–91^. To translate cellular reprogramming methods to the clinic, efforts are currently mainly focused on direct reprogramming of human cardiac cells. Previous studies found that the combined expression of the transcription factor c-ETS2 and mesodermal posterior protein 1 (MESP1) transformed human dermal fibroblasts into cardiac progenitor cells that express early cardiac factors, such as protein ISL1 and homeobox protein NKX2-5. This effect was not detected in directly reprogrammed mouse fibroblasts (Fig. 3)^92^.

## Immune cells in the heart

Immune cells play important roles in heart of steady state, an increasing number of studies on cardiac injury repair also have shown that the various immune cells that reside or infiltrate the heart tissue play roles in the process of injury repair. Immune cells identified in heart, including macrophages, monocytes, neutrophils, dendritic cells (DCs), T cells and B cells, eosinophils, mast cells, which can reside or infiltrate cardiac tissue^93^ to maintain cardiac function.

### Immune cells in cardiac homeostasis

#### cTMs phagocytose bacteria and apoptotic cells

Macrophages typically maintain tissue homeostasis and tissue repair, promote angiogenesis and phagocytosis of apoptotic cells and necrotic cells, clear and kill bacteria, and produce proinflammatory cytokines^94^. Macrophages are present in all tissues and participate in tissue growth and remodeling from the earliest stages of development^95,96^. Like in most tissues, macrophages are the main immune cells that reside in the heart. They are usually found near endothelial cells or in the interstitial space^95,97^. Cardiac tissue resident macrophages (cTMs) are spindle-shaped cells located in the interstitial space between muscle cells, fibroblasts and endothelial cells (ECs)^98^. cTMs have been widely characterized as an integral part of organ development. It has been shown that cTMs originate from the yolk sac early in development and are maintained until adulthood in mouse models^99^. cTMs have CCR2-MHC-low cell surface characteristics and are closely related to blood vessels in the myocardial wall during embryonic wall development, and their absence causes coronary vascular malformations. cTMs are located throughout cardiac tissues and are closely related to blood vessels to regulate the supply and discharge of blood. cTMs are also abundant in the cardiac conduction system^97,98^. In the steady state, cTMs phagocytose subcellular particles containing dysfunctional mitochondria ejected from CMs, which supports CM health^100^. In addition, cTMs help heart-specific processes and participate in maintaining proper electrical conduction^98^. A large number of cTMs present in the AV node are connected to cardiomyocytes through connexin 43 (CX43), and the destruction of Cx43 delays the electrical conduction of the AV node. To maintain homeostasis, cTMs interact closely with ECs and quickly internalize blood-borne fluorescein isothiocyanate dextran (FITC-dextran). cTMs in the adult heart phagocytose bacteria and apoptotic cells, indicating that cTMs have typical macrophage characteristics^95,101^. Many macrophages utilize macropinocytosis and phagocytosis to take up components of the local microenvironment. At the cellular physiological level, cTMs phagocytose fluorescently-labeled bacteria, indicating the ability of these cells to phagocytose bacteria^101^. The cTMs in the cardiac tissue are located between CMs and phagocytose some molecules produced by the surrounding CMs and absorb dead CMs^95,97^. cTMs also produce nutritional and immune-related factors and make contact with CMs to participate in the regulation of cardiac homeostasis^102^.

Monocytes and macrophages appear to play an important role in cardiac renewal. Macrophages have multiple roles in normal and injured hearts. cTMs are different from monocytes isolated from the adult mouse spleen and brain, and their gene expression profile is similar to that of anti-inflammatory M2 macrophages^103^. After gene ablation in adult mouse CMs, M2-like cTMs were replaced by macrophages derived from proinflammatory monocytes^6^. In a healthy state, macrophages express a large number of M2-specific markers^97,104^. This expression is also consistent with the characteristics of M2 macrophages, because M2 macrophages usually promote tissue reconstruction after injury, thereby helping to restore a steady state. cTMs are the most abundant cell populations in heart, they respond to injury signals by initiating neutrophil recruitment^105^ and secreting beneficial tissue and immunoregulatory factors^97^. Therefore, cTMs play an important role in maintaining cardiac homeostasis in steady state.

#### NK cells have an inhibitory effect on some cardiac diseases

NK cells are a large subset came from the innate lymphoid cell (ILC) family. The special markers expressed in mature mouse NK cells are DX5 and CD11b, there are also along with various organ-specific chemokine receptors on cell surface, and exhibit cytokine secretion profiles and inherent cytotoxic abilities that may be beneficial to migrate from the bone marrow to specific sites in the body^106,107^. NK cells are dysregulated in some cardiac diseases, including myocarditis, transplant rejection, and cardiac fibrosis, such as in a mouse model that limited myocarditis, NK cells are essential for suppressing cardiac viral infection and replication and reducing cardiac eosinophil infiltration. NK cells prevent the maturation and transport of inflammatory cells, which alter the environments of local cytokines^108–110^, such as IFNγ and other mediators, which are produced by NK cells, and induce apoptosis in nearby resident and hematopoietic cells.

#### DCs have a protective effect

DCs exist in the spleen and other lymphatic and nonlymphoid tissues and mainly serve as antigen-presenting cells. DCs are divided into two subtypes: cDCs, which exist in lymphoid tissue, blood and nonlymphoid tissue, and pDCs, Cardiac cDCs are divided into cDC1s and cDC2s according to their dependence and the expression of different transcription factors (TFs). Cardiac cDC1s express the markers CD103, CADM1, XCR-1, DNGR-1 (Clec9a) and IRF8, Similar to cDC1s in other organs. knockout of the transcription factor IRF8 in CD11c + cells in mice, cDC1s lack in all organs, including the heart^111,112^. DCs in the heart can produce different cytokines, such as tumor necrosis factor (TNF-α), IL-12 and IL-23, all of which have specific functions^113,114^. DCs upregulate cardiomyocyte hypertrophy and inflammation-related genes induced by advanced glycation end products (AGEs)^115^. Compared with cDCs in most other tissues, cardiac cDCs recruit to the heart by the chemokine receptor CCR2 without local proliferation^116^. Cardiac cDC1s in healthy heart migrate to draining mediastinal lymph nodes, to present cardiac autoantigens to specific αMyHC- CD4 + T cells, resulting in regulatory T cell (Treg cell) expansion. Compared to cDC2s phenotypes in other organs, cardiac cDC2s express the markers CD172α and CD11b and the transcription factor IRF4^117^. Although the heart is not in direct contact with the external environment, histological studies conducted on several nonlymphatic rat tissues in 1981 proved for the first time that the number of DCs in healthy hearts was comparable to that in barrier tissues such as skin^118^. It has been proposed to use cardiac DCs as gatekeepers against invasive heart disease and self-tolerance^118^.

### Immune cells in cardiac injury repair

The response of immune cells to cardiac injury repair is divided into three overlapping phases: inflammation, proliferation, and maturation. Initially, resident and infiltrating immune cells activate inflammation and proliferation, and the relative balance between pathological inflammation and proliferation determines the development of HF^119^. The kinetics of the inflammatory and proliferation phases influence the repair results. Studies have shown that resident and recruited immune cells play roles in cardiac injury and appear in cardiac tissue in the early stage of disease^120–124^.

Immune cells embedded in injured heart tissue recognize and regulate inflammation by dynamically interacting with stromal cells in the interstitial heart, which may lead to the reappearance of heart morphology to support regeneration by rebuilding functional scaffolds in regenerative organisms or fail to resolve the inflammatory response and produce fibrotic scar tissue in adult mammals^125^. Immune cell stimulation is one of the earliest reactions that can be detected at the infarct site after MI, and the immune response plays an important role in coordinating multiple processes that control cardiac repair, including the survival of resident cells, the removal of fibrotic and dead cells, infarct area formation and vascular reconstruction. In fact, the early stage of inflammation is characterized by rapid sterile inflammation, immune cell infiltration and phagocytosis to remove damaged cells and extracellular matrix tissues, followed by the proliferation stage, processes that muscle layer fibroblasts proliferation, scar formation and neovascularization subside in the next few days^126^. Neutrophils, monocytes, endothelial cells and pericytes help to suppress and eliminate inflammatory responses. In addition, changes in the composition of the extracellular matrix are also involved in the suppression of inflammatory signals^126^. However, excessive infiltration of the myocardium by inflammatory cells exacerbates cardiac injury and worsens myocardial remodeling after MI through the release of proinflammatory cytokines, cytotoxic mediators, and reactive oxygen species (ROS)^127–129^. The recruitment of inflammatory cells must be strictly controlled to ensure tissue healing while avoiding excessive inflammatory responses after injury, which can otherwise lead to poorly adaptive remodeling and contractile dysfunction^130^. Cardiomyocytes secrete various anti-inflammatory signals, such as cytokine 1, MIC1, and growth differentiation factor 15 (GDF-15, which inhibits macrophages), to limit the infiltration of immune cells and abolish chronic inflammation.

#### cTMs promote CM proliferation and angiogenesis

After cardiac injury, cTMs are thought to play a key role in heart remodeling during the inflammatory phase. cTMs, especially the MHC-II-low subgroup, phagocytose dying CMs, contributing to local homeostasis. After myocardial injury, the activation of inflammatory bodies has weak tissue regeneration, and blockade of the CCR2 axis prevents ischemic injury^131,132^. After Ang II infusion, cTMs expanded without peripheral monocyte proliferation in situ, similar to the expansion of pleural macrophages after worm infection^133^.

cTMs regulate cardiac progenitor cell proliferation, especially in during the proliferation phase after heart injury. A genetic mouse model of myocardial loss was used to demonstrate that newborn mice have expanded populations of embryonic-derived cTMs, resulting in minimal inflammation and promoting cardiac recovery through the proliferation of myocardial cells and angiogenesis^6^. Using the macrophage depletion model, cardiac regeneration and neovascularization after MI were shown to depend on neonatal macrophages. Newborns lacking macrophages do not regenerate cardiac muscle and form fibrotic scars, resulting in reduced cardiac function and angiogenesis^5^. Macrophage depletion weakens the regenerative capacity of newborn animals and young mammals, highlighting the key role of macrophages in tissue repair after injury^5,134^. In addition, after systemic macrophage depletion, cardiac inflammation or senescence, CCR2 + Ly6C-high monocytes replace embryonic resident macrophages and coordinate heart inflammation^95,135^.

cTMs perform differently in the heart in different phases. In the neonatal heart, cTMs are essential for cardiac regeneration after MI injury, probably because cTMs have a phenotype of polarization and secrete necessary soluble factors that drive angiogenesis^5^. However, this protective mechanism disappeared within 2 weeks, approximately when monocyte-derived macrophages were recruited to the site of heart injury^6^. In the aging heart, the macrophage phenotype converts to the proinflammatory subtype, leading to inflammation^136^.

#### NK cells prevent cardiac fibrosis and promote vessel remodeling

NK cells play an important role in regulating the proliferation phase after cardiac injury. In the proliferation phase, NK cells prevent the development of cardiac fibrosis by directly restricting collagen formation of cardiac fibroblasts and the accumulation of specific inflammatory populations and profibrotic cell types such as eosinophils in the heart^137^. NK cells activated by IL-2 injection promote blood vessel remodeling through α4β7 integrin and killer lectin-like receptor subfamily G member 1 (KLRG1) without participating in the basic formation of blood vessels. Activated NK cells first bind to cardiac epithelial cells (CECs) through α4β7 integrin and vascular cell adhesion molecule 1 (VCAM-1) and disrupt the binding of N-cadherin through KLRG1. This process transfers β-catenin from the cytoplasm to the nucleus and eliminates the inhibitory effect of cell contact on proliferation^138^. In addition, NK cells interact with cardiac endothelial cells to increase vascularization and angiogenesis after MI^138,139^.

#### M1 stimulates myocardial fibrosis and then M2 promotes infarct scar formation and angiogenesis

Recruitment but not local proliferation is the main mechanism by which the number of monocytes and macrophages is regulated in the ischemic myocardium^140^. Macrophages in the hearts of adult mice are a heterogeneous population. In the heart, there are different subsets of macrophages with different functions and origins, including protective and pathogenic cells^103^. Injured cardiac tissues after MI or I/R have obvious myocardial macrophage and neutrophil infiltration^141^. After cardiac tissue injury, monocytes quickly mobilize from the bone marrow to the injured tissues, where they differentiate into macrophages or DCs and trigger an immune response. In MI, monocytes released by the spleen are recruited to the heart through the MCP-1/CCR2 interaction. When monocytes are recruited into cardiac tissue, these cells differentiate into inflammatory M1 macrophages and activated M2 macrophages^142^. Due to their abundance and phenotypic plasticity, macrophages are well suited to coordinate the repair response after MI. Macrophages have almost unlimited potential, and macrophage subpopulations are regulated to mediate protection of cells in the infarcted heart and are used to activate survival, repair, and regeneration responses^143^.

M1 macrophages are proinflammatory and usually induced by IFN-γ or TNF-α, while M2 macrophages are anti-inflammatory and usually induced by IL-4/IL-13/IL-10^97,104^. M1 macrophages are mainly in the early stage after MI. Infiltrating monocyte-derived M1 macrophages trigger the cardioprotective phenotype in the ischemic heart and play a positive role by activating antiapoptotic programs in cardiomyocytes^143^. A large number of M1 macrophages with proinflammatory characteristics are rapidly recruited to cardiac tissues, and M1 macrophages stimulate myocardial fibrosis by inducing the production of TGF-β1, while TGF-β1 stimulates Smad3 signaling, induces the production of collagen and MMP, and releases extracellular matrix from cardiac fibroblasts to promote tissue fibrosis and myocardial remodeling^144^. In this situation, Treg cells are important regulators of macrophage phenotype and function^145–147^. After ingesting dead cells and matrix fragments, macrophages release anti-inflammatory cytokines, which suppress inflammatory damage and reduce undesirable cardiac remodeling^148^. During the proliferation phase, M2 macrophages are the mainly subpopulations, which promote cardiac remodeling through infarct scar formation and angiogenesis. By inhibiting the expression of IL-6, MMP9, TNF-α and producing exogenous signals such as IL-10, macrophages are transformed into a repair-mediating M2 phenotype^93^. The result of this transformation is the emergence of M2 macrophages that produce IL-10, TGF-β and VEGF, thereby promoting fibrosis and angiogenesis, as well as the production of other factors, such as myeloid-derived growth factors^126,149^. Following the maturation phase, the wound begins to subside, and the infiltrating macrophages show features associated with inflammation inactivation/reduction, which promotes cardiac repair by mediating the fibrotic response^150^.

During the stage of infarct healing, monocytes/macrophages with strong phagocytosis abilities clear dead cells and matrix fragments; during this process, the scavenger receptor cluster protein 36 (CD36) can be detected on cardiac LY6C-high monocytes, and CD36 is essential for early phagocytosis and small infarct size of dying cardiomyocytes after permanent coronary ligation^151^. Hepcidin inhibits macrophage-induced cardiac repair and regeneration by regulating the IL-4/IL-13 pathway^152^. Overexpression of monocyte MCP-1 in the heart induces macrophage infiltration, new blood vessel formation, proliferation of myocardial fibroblasts and IL-6 secretion, which leads to the prevention of LV dysfunction and remodeling after myocardial infarction^153^. Early interference with macrophage recruitment is sufficient to disrupt the formation of new blood vessels, impeding neutrophil clearance and cardiac regeneration. In the mouse MI model, myocardial cells secrete REG3β to recruit macrophages after injury, and the loss of REG3β results in a large decrease in the number of ischemic cardiac macrophages, accompanied by insufficient neutrophil clearance and increased ventricular expansion^154^. Similarly, the loss of REG3β also leads delayed and reduced macrophage recruitment of the heart in a teleost medaka after injury and delayed neutrophil clearance. Observations obtained from mice, zebrafish, and medaka indicate that cardiac repair and regeneration require the timely recruitment of macrophages^155^.

The recruitment of M1 macrophages is triggered by a mechanism that partially involves CM-derived BMP4 in CM-conditioned medium. It has also been found that M1 macrophages affect the proliferation and differentiation potential of CMs, partly because of BMP molecules secreted by macrophages. BMP molecules involve the activation of classic inside-out signaling pathways, such as Smad1, 5, and 8, which are known to be activated during myocardial injury in vivo^156^. Angiotensin II (ANG II) is upregulated in the serum and cardiac tissue of mice with experimental autoimmune myocarditis (EAM), and ANG II significantly drives monocyte/macrophage recruitment through the CC chemokine receptor 2/5 (CCR2/5) axis. Two cytokines, IL-4 and IL-13, control the expression of chemokines^157^. Monocyte/macrophage infiltration is eliminated and development of EAM is improved by CCR2/5 antagonists and ANG II receptor inhibitors^158^. The macrophage gene expression pattern also changes when the myocardium is remodeled. Compared with that of the steady state, the expression of metalloproteinases in monocyte-derived macrophages and resident macrophages during HF is reduced, which may affect matrix renewal. More IL-1β is produced by monocyte-derived macrophages during HF than during normal conditions, while resident macrophages contribute more TNF. However, monocyte-derived macrophages also produce VEGF^159^, which may be related to cardiac hypertrophy-induced angiogenesis.

#### Neutrophils lead to cardiac damage early, but also promote cardiac injury later

CCR2 + monocytes and macrophages residing in the heart are essential for promoting neutrophil infiltration into injured myocardial tissue^160^. Necrotic cardiomyocytes lead to the activation of tissue-resident immune and nonimmune cells. Neutrophils are innate immune cells that migrate to damaged lesions within 24 h of cardiac injury in large numbers^161,162^. Neutrophils the first type of immune cell to infiltrate the infarcted myocardium and are also the first type of immune cell to be recruited to the myocardium in large numbers after ischemic cardiac injury or pressure overload^8^.

Sterile inflammation occurs when tissues are injured after infection. Neutrophils are recruited from the blood to inflammatory sites^163^, because they have pro-inflammatory and cytotoxic properties, they not only promote wound healing but also cause tissue damage^164^. Studies have found that the recruitment of neutrophils occurs after focal hepatic necrosis. The main mechanism is that adenosine triphosphate released by necrotic liver cells activates the Nlrp3 inflammasome, and the resulting inflammatory microenvironment promotes neutrophils adhering to the sinusoids^165^. Subsequently, the concentration of chemokines in the blood vessel changes, under the chemotaxis of these chemokines, neutrophils migrate from healthy tissues to foci of damage^166,167^.Inflammation induced by neutrophils initiates myocardial repair during MI^127^. In the early stage, macrophages and neutrophils secrete proinflammatory cytokines, which promote fibroblast differentiation and lead to sustained inflammation and myocardial injury^168^. The recruitment of neutrophils increases inflammation and leads to cardiac dysfunction.

This inflammatory response includes a series of events. First, neutrophils and monocytes/macrophages infiltrate to remove debris from necrotic cells by releasing high levels of ROS and secreting proteases and proinflammatory mediators, which not only induce tissue damage but also further recruit leukocytes; for example, neutrophils support the recruitment of proinflammatory LY6C-high monocytes and dominate between day 1 and day 4 after injury, maintaining inflammation and removing cellular debris^169^. Then, a proliferation phase follows the initial inflammatory phase, which is characterized by the expansion of neutrophils and macrophages, these cells remove dead cells and matrix fragments, as well as release cytokines and growth factors that can form highly vascularized structures consisting of connective tissues and new blood vessels and eventually heal tissues. The proliferation phase is characterized by fibroblast activation and endothelial cell proliferation, which ultimately leads to repairable myocardial fibrosis, angiogenesis, extracellular matrix deposition and growth factor release. Finally, inflammation and scar formation are resolved^169^. Defects in neutrophils lead to delayed collagen deposition, enhanced matrix degradation and increased sensitivity to heart rupture^154^. Therefore, neutrophil activation must be strictly controlled, although neutrophils are essential for initiating an acute inflammatory response^170^.

Neutrophils are recruited to the heart, and the response factors include DAMPs, cytokines including chemokines, endogenous lipid mediators (such as prostaglandin E2 and leukotriene B4), histamine and components^171,172^. The recruitment of neutrophils to the site of infection depends on CXC chemokines^173^. In mice, CXC chemokines control the recruitment of neutrophils through CXCR2, while in humans, neutrophil recruitment depends on both CXCR1 and CXCR2^174^. Neutrophils produce cytokines and chemokines, which attract spleen-derived macrophages to migrate into cardiac tissue^175^.

As regulators of immune responses, neutrophils affect chronic immune responses and the function of dendritic cells and lymphocytes. Neutrophils have been shown to improve the polarization of macrophages toward a repair phenotype by releasing lipoproteins associated with neutrophil gelatinase, thereby improving heart healing after MI^126,176^. The depletion of neutrophils does not affect the infarct size but worsens cardiac function and HF and increases cardiac fibrosis^176^.

#### DCs are immunoprotective in the heart

DCs are protective immunoregulators in post-MI healing^177^. During the healing process after MI, BM-derived activated CD11c + CD11b + DCs penetrate into the infarcted heart. It has been reported that after MI, DCs accumulate early in the infarct border area in rats and mice, and the number of cells reaches a peak at the 7th day^177,178^. DCs mediate the regulation of monocyte and macrophage homeostasis after MI, which indicates that DCs are beneficial to cardiac injury and repair. After activation, DCs recruit to lymphoid organs, where they accumulate and stimulate T cells^179^. The myocardium contains both pDCs and cDCs, which are necessary for maintaining the infiltration of congenital leukocytes^180^.

After MI, only cDCs participate and play a key pathological role^181^. By depleting cDCs exclusively, it has been demonstrated that the measured immune response and infiltration of macrophages, neutrophils and multiple T cell subpopulations are blunted, which are related to the indicators of improvements in cardiac structure and function. The depletion of cDCs is related to reduced mRNA expression of the proinflammatory cytokines IL-1β and IFN-γ in the heart^182^. In vivo analysis showed that in CD11c + DC-deficient mice, left ventricular function deteriorated after MI. After depleting bone marrow-derived DCs in CD11c-Bifidobacterium toxin receptor transgenic mice, left ventricular function and impaired remodeling were worsened after MI^183^. The DC depletion group showed long-acting inflammatory cytokines, such as IL-1β, IL-18 and TNFα. In addition, in the hearts of the DC depletion group, anti-inflammatory Ly-6C-low monocytes and other activated macrophages were also significantly infiltrated^177^. These results indicate that cardiac DCs have a strong immunoprotective function after MI. DCs prevent tissue-destructive autoimmunity after heart injury by activating conventional fork-head box protein P3 (FOXP3)– CD4 + T helper cells and FOXP3 + CD4 + Treg cells^184^. A reduction in the number of DCs is associated with heart rupture after MI, increasing the recruitment of proinflammatory monocytes that maintain the production of proinflammatory cytokines^185^. In short, DCs protect the heart by regulating the recruitment of different types of immune cells.

#### NKT cells protect the heart by producing IL-10

**Fig. 4:**
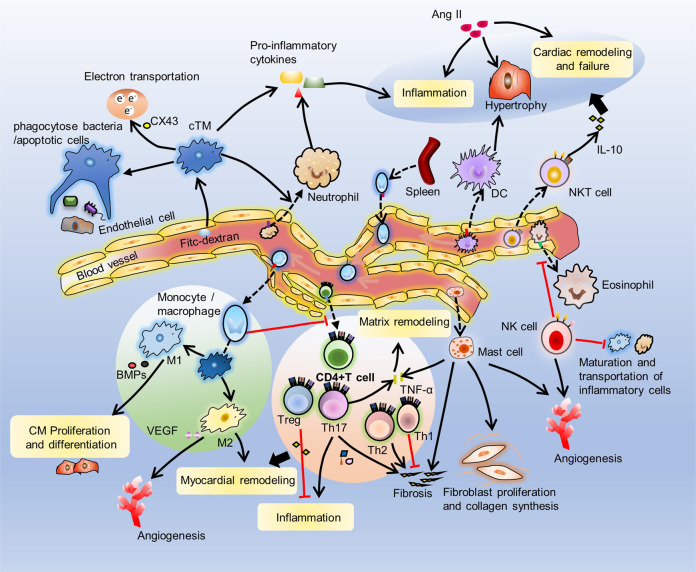
Immune cells in the heart. cTMs internalize blood-borne FITC-dextran; cTMs also have typical macrophage characteristics and phagocytose bacteria and apoptotic cells. cTMs regulate electrical conduction through CX43, and cTMs can also produce proinflammatory cytokines to induce inflammation in the aging heart and promote neutrophil infiltration. Resident NK cells reduce cardiac eosinophil infiltration. NK cells prevent the maturation and transport of inflammatory cells. Monocytes are recruited into cardiac tissue and differentiate into M1 and M2 macrophages. M1 macrophages affect the proliferation and differentiation of CMs by BMPs, and M2 macrophages are related to angiogenesis by producing VEGF. Recruited neutrophils secrete proinflammatory cytokines, which promote fibroblast differentiation and lead to sustained inflammation. DCs upregulate cardiomyocyte hypertrophy a. NKT cells secrete cytokines such as IL-10 to protect or regulate hypertrophy, cardiac remodeling and the inflammatory response induced by Ang II. Infiltrating CD4 + cells include helper T cells (Th1 and Th2), Th17 cells and Treg cells. Th1 cells reduce the fibrotic response, while Th2 and Th17 cells promote fibrosis. Th17 cells also promote inflammation and extracellular matrix remodeling, and Treg cells reduce inflammation. Mast cells promote fibrosis, angiogenesis and cardiac fibroblast proliferation and collagen synthesis through TNF-α

Natural killer T cells (NKT) are unique T lymphocyte subsets characterized by coexpression of NK receptors and unchanged T cell receptors (TCRs). TCRs recognize glycolipids presented by the major histocompatibility complex (MHC);^186,187^ these cells secrete T helper type 1 (Th1), Th2 or immunoregulatory cytokines^188–190^ such as IL-10 to modulate immune responses towards proinflammatory or regulatory profiles^191,192^. NKT cells involve in inflammation and tissue remodeling. These cells play a protective role in left ventricle (LV) remodeling and failure. After MI in mice, the recruitment of NKT cells in the non-infarcted area of the LV increased^193^. Pressure overload induced by transverse aortic constriction also increases the infiltration of invariant natural killer T (iNKT) cells in mouse hearts^194^. LV remodeling and the transition from hypertrophy to heart failure are exacerbated after the disruption of iNKT cells, and this process is associated with the activation of mitogen-activated protein kinase signaling^195^. The class I antigen-presenting molecule CD1d is mainly expressed on antigen-presenting cells, which combines to TCRs in NKT cells^191^. CD1d deficiency significantly accelerates Ang II-induced hypertrophy, causing cardiac remodeling and an inflammatory response^196^. DCs lacking CD1d reduce IL-10 production by NKT cells, and the administration of IL-10 to CD1d-KO mice may significantly reverse Ang II-induced hypertension and cardiac remodeling by activating STAT3 and inhibiting the TGF-β1 and NF-kB signaling pathways^197,198^. In addition, administration of the NKT cell activator α-galactosylceramide (α-GC) in the mouse myocardial infarction model resulted in enhanced infiltration of NKT cells in the non-infarcted area^199^, and it was found that α-GC administration significantly reduced left ventricle expansion and mortality caused by HF. It is suggested that these effects depend on NKT cells, and IL-10 is the most effective effector cytokine in this process (Fig. 4).

#### T cells promote heart remodeling through cytokines and growth factors

Antigen-activated T cells are recruited more efficiently than monocytes to infected cardiomyocytes, and early recruitment of effector T cells may also be involved in the maintenance of inflammatory cells at the site of chronic local infection^200^. The depletion of monocyte/macrophage lineage cells in the hypertensive heart leads to massive infiltration of inflammatory cells, mainly CD4 + T lymphocytes, in the area of cardiomyocyte loss^201^. These results suggest that monocytes/macrophages have a protective effect on adaptive immunity by inhibiting T cell recruitment of the heart. Antigen-activated CD4 + T cells are divided into four subtypes, including helper T cells (Th1 and Th2, Th17 cells) and Treg cells.

T lymphocytes have different roles in cardiac tissue injury and repair. The traditional view is that myocardial scar formation after MI only represents fibrosis with extracellular matrix proteins replacing necrotic tissues. Unlike the traditional view, it is now very clear that T cells are effective in coordinating this process. The resulting fibrosis indicates that T cell infiltration plays a central role in the pathogenesis of cardiac fibrosis^202^. Several experimental animal models have shown T cell regulatory mechanisms involving immune cell tolerance to cardiac antigens, including processes of T cell activation, recruitment of T cells to the heart, and cytokine production in response to ischemic or nonischemic sterile inflammation^203^. At present, the related mechanism of T cell recruitment of the heart involves c-Met signaling-mediated induction of the release of autocrine CCR5 ligand, which promotes the recruitment of T cells to the heart through the chemokine receptor CCR5^204^. The mechanism of T cell recruitment and myocardial infiltration in the MI experimental animal model seems to be time-dependent. For example, in myocarditis, the time after ischemia is different and may involve different responses. Primary myocarditis is only a small part of the cause of human HF, but in this case, the T cell infiltration mechanism is still observed in the more common form of HF, and T cells have a broad role in promoting cardiac fibrosis and failure.

In a mouse model of permanent coronary infarction, infiltrating CD4 + and CD8 + T cells gradually infiltrate the heart and reach a peak on the 7th day after MI. The infiltration of T cells into the myocardium may directly regulate the phenotype and function of fibroblasts at all stages of this process. In cardiac tissue, antigen-driven expansion of oligoclonal T cells may be an effector of rheumatic cardiac disease. Generally, Th1 cells secrete Th1 cytokines (such as IL-2, IFN-γ, and TNF-α) and promote the antifibrotic response in cardiac tissue, while Th2 cytokines (such as IL-4, IL-5, and IL-13) promote cardiac tissue fibrosis^205^. Th17 cells are CD4 + T cells that produce IL-17 and IL-22, play an important part in promoting inflammation during the cardiac tissue remodeling. Th17 cells assist in host resistance to infection by recruiting neutrophils and macrophages into infected tissues^206^. Th17 cells induce or enhance the expression of inflammatory cytokines, such as IL-6, IL-1β and TNFα, and promote extracellular matrix remodeling by producing repair-associated components such as matrix metalloproteinases (MMPs) or proteoglycans^207^. In addition to their effects on the early stages of infarct scar formation during chronic remodeling, Th17 cells in the heart seem to guide the distal fibrosis and scar formation that occur throughout the left ventricle of the heart^202^. In the rat model of HF induced by intraperitoneal injection of isoproterenol, the use of anti-IL-17 antibodies to block the production of IL-17 leads to a reduction in cardiac fibrosis. In this model, the expression of MMP-1 and receptor activator of NF-κB ligand (RANKL) and collagen synthesis in cardiac fibroblasts are inhibited, but MMPs and osteoprotegerin (OPG) are increased in tissue^208^. These results indicate that Th17 cells regulate cardiac fibrosis by the production of MMPs through the IL-17-RANKL/OPG system in cardiac fibroblasts, or by stabilizing the mRNA of proinflammatory cytokines in various cardiomyocytes and immune cells.

Treg cells are CD4 + CD25 + subpopulations of T lymphocytes that have strong inhibitory properties. Treg cells, as inhibitors of inflammation after MI, play an important role in immune homeostasis. Under physiological and pathological conditions, Treg cells both play a key role in control of innate and adaptive immune responses^209^. There are chronic inflammation appearing in adverse ventricular remodeling after MI^210^, almost all processes related to ventricular remodeling such as degradation of the interstitial matrix and collagen deposition, hypertrophy and apoptosis of the remaining cardiomyocytes, scar formation, and ventricular dilation are associated to inflammation^211^, Treg cells have beneficial effect on undergoing ventricular remodeling by CD28 superagonist-mediated expansion and adoptive cell migration after MI^212^. In the inflammatory phase, Treg cells protect the heart from adverse ventricular remodeling and maintain cardiac function by inhibiting proinflammatory cell infiltration and directly protecting cardiomyocytes^212^. In mice, Treg cells may reduce the inflammatory response during chronic cardiac remodeling after MI by inhibiting self-effector T cells, and effector T cells are controlled by the expansion of Treg cells. In the proliferation phase, Treg cells have been shown to directly activate and promote local progenitor cell regeneration^213,214^, and Treg cells promote myocardial recovery by the IL­10-dependent pathway^215,216^. In zebrafish, Treg cells enhance precursor cell proliferation by activating neuromodulin-1 in the heart through IL-10 secretion. Treg cells indirectly regulate cardiac regeneration by controlling neutrophils^217–219^ and inducing macrophage polarization^5,6,220^.

Treg cells are also thought to be involved in M2 polarization after MI by producing IL-10^221^. In cardiac disease models, ANG II infusion and MI increase the number of CD4 + CD25 + Treg cells. Adoptively transferred Treg cells reduce cardiac hypertrophy, inflammation and fibrosis through IL-10 production and direct cell-cell interactions^211,222^. These results indicate that there is a close relationship between Treg cells, CMs and cardiac fibroblasts, which confirm the importance of Treg cells in cardiac remodeling.

The absence of effector T cells can increase the recruitment of proinflammatory monocytes and reduce neovascularization and collagen deposition^184^. The loss of IFN-γ expression in the injured myocardium due to insufficient Treg cell recruitment may affect other immune cell populations, including macrophages and neutrophils^223–225^.The depletion of Treg cells triggers autoimmunity and cardiac dysfunction and enhances the immune response to nonself antigens^226^. MHC class II-deficient mice and mice expressed a single transgenic T cell receptor show impaired wound healing and monocyte population expansion after ischemic injury. This finding indicates that self-antigens are presented to CD4 + T cells through MHC class II-expressing cells, such as macrophages and DCs, which then drives the immunosuppressive response in the myocardium^125^.

#### B cells regulate wound healing and tissue remodeling

Both T cells and B cells can regulate wound healing and tissue remodeling after myocardial injury. Effector T cells are activated in the proximal lymph nodes and quickly colonize the injured heart after MI^227^. First, while the number of B cells peaks after the onset of ischemia^228^, B cells produce proinflammatory cytokines to reduce cardiac contractility and promote cardiomyocyte apoptosis^229^; then, B cells accumulate in the infarcted heart, produce IL-10 affected by pericardial adipose tissues (PAT) and preferentially express cytokines, and B cells promote inflammation resolution and reduce myocardial injury to preserve cardiac function during the resolution of inflammation after MI. The specific loss of B cell-derived IL-10 after MI worsens cardiac function, exacerbates myocardial damage, and delays inflammation remission^230^.

#### Eosinophil recruitment by CCR3 and its ligands CCL11, CCL24, and CCL26

Eosinophils may play a pathogenic role in myocarditis. In mouse models of myocarditis and the hearts of patients with eosinophilic myocarditis, the recruitment of eosinophils to the injured heart depends on the expression of the chemokine receptor CCR3 and its ligands CCL11, CCL24, and CCL26. Cardiac fibroblasts are the source of CCL11 in the interstitium of the heart. CCL24 is produced by F4/80+ macrophages located at the focal foci of the heart. The expression of CCL11 and CCL24 is controlled by the cytokines IL-4 and IL-13 produced by Th2 cells. These findings are currently known as the precise pathway for eosinophil recruitment to the heart, and blocking this pathway prevents eosinophil-mediated heart injury^157^.

#### Mast cells induce myocardial remodeling and cardiac fibrosis

Mast cells (MCs) mainly regulate allergic reactions and host-pathogen interactions in the innate immune system, and myocardial MCs are known to infiltrate and proliferate in MI, arrhythmia, atherosclerosis, HF, and cardiomyopathy.

**Fig. 5:**
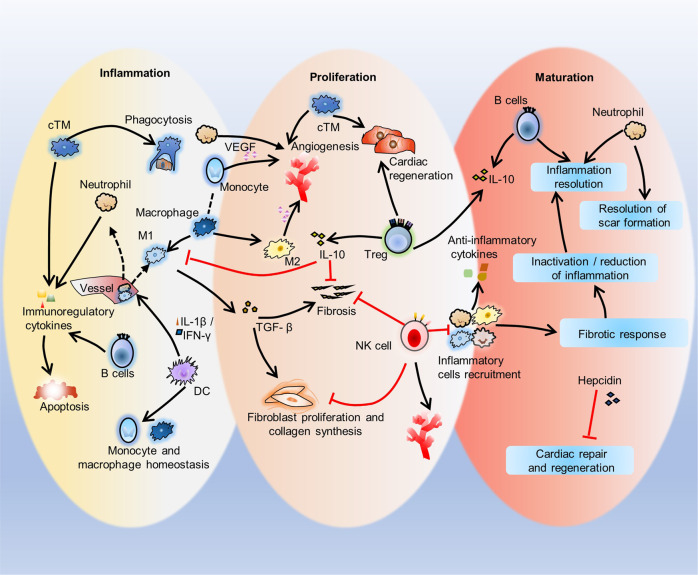
Three phases of immune response to cardiac injury repair. This diagram shows the inflammation, proliferation, and maturation phases after cardiac injury. In the Inflammatory phase, cTMs phagocytose dying cardiomyocytes. B cells secrete immunoregulatory factors to reduce cardiac contractility and promote cardiomyocyte apoptosis. DCs mediate the recruitment of inflammatory cells such as monocytes and M1 macrophages and homeostasis. In the proliferation phase, cTMs promote the proliferation of myocardial cells and angiogenesis. Neutrophils, monocytes and M2 macrophages also promote angiogenesis though VEGF, and M1 macrophages promote tissue fibrosis and myocardial remodeling by inducing extracellular matrix release from cardiac fibroblasts. NK cells protect against cardiac fibrosis by directly restricting collagen formation of cardiac fibroblasts and preventing the accumulation of specific inflammatory populations, and NK cells also promote blood vessel remodeling. Treg cells inhibit inflammation and fibrosis and promote precursor cell proliferation and macrophage polarization. In the maturation phase, the recruitment of inflammatory cells such as macrophages, neutrophils and eosinophils is inhibited, anti-inflammatory cytokines are secreted, infiltrating immune cells regulate inflammation inactivation/reduction by mediating the fibrotic response, and inflammation and scar formation are resolved. During this phase, hepcidin inhibits macrophage-induced cardiac repair and regeneration

The number of MCs also increases when human cardiac muscle is hypertrophic^231^. These cells secrete some molecule such as platelet-derived growth factor A (PDGFA), TNF-α, TGF-β and histamine, which affect heart function. MCs are the main source of TNF-α and have different production methods. TNF-α induces MMP activation, leading to myocardial remodeling^232^. After pressure overload in the mouse heart, recruited mast cells induce PDGFA chain synthesis and promote cardiac fibroblast proliferation and collagen synthesis. In addition to PDGFA chain, ANG II-induced Rac 1 activation also leads to myocardial remodeling and atrial fibrillation through CTGF and lysyl oxidase-mediated miR-21 expression^231^. In myocardial ischemia and MI, local MCs release some mediators which contribute to coronary vasoconstriction, leukocyte recruitment, formation of new blood vessels, scar formation^233^. Among the mediators released by MCs, histamine and ET-1 promote severe arrhythmias, leading to sudden cardiac death^234^. In addition to coronary vasoconstriction and systolic failure, arrhythmias, including symptoms such as arrhythmia, premature beats, and atrial fibrillation, are also features of cardiac allergic reactions, which are caused by the release of several mast cell mediators^235^. In coronary atherosclerosis, mast cells release mast cell tryptase, chymotrypsin, and TNF-α to promote cholesterol accumulation and plaque instability^236^. Infiltrating MCs in the allergic dead heart are significantly increased, and the serum level of mast cell tryptase is high and is accompanied by severe pulmonary congestion and edema^237^. MCs are recruited and activated in the drug-related dead heart. The degree of MC degranulation, increase in tryptase levels and pathological changes in the victim’s heart related to the drug are similar to those of allergic death^238^. In HF, mast cell chymotrypsin causes progressive LV dysfunction by promoting cardiomyocyte apoptosis and fibroblast proliferation^239^. Chymotrypsin and tryptase also promote the characteristic fibrosis associated with cardiomyopathy. Myocardial remodeling and hypertrophy are induced by the release of cardiac MC mediators and proteases are typical characteristic of advanced HF associated with hypertension. In addition, recruited cardiac MCs contribute to fibrosis associated with autoimmunity and viral myocarditis^240^. MC-stabilizing drugs improve HF by reducing myocardial remodeling. Atrial fibrosis requires MCs to interact with fibroblasts in the heart and affects the sensitivity of atrial fibrillation, which is the most common type of arrhythmia in HF^241^. These results indicate that cardiac MCs play a key role in allergic cardiac diseases through certain mediators, such as TGF-β, TNF-α and histamine, and MCs also regulate atrial myocardial remodeling and communication between CMs and cardiac fibroblasts (Fig. 5).

## Conclusion

There are four main types of cardiac tissue injury: ischemia-reperfusion injury (IRI), cryoinjury, resection, and gene ablation. Many studies have used IRI as a pathological model of MI. At present, there are two types of therapy for heart injury repair. One type of therapy functions in the molecular level. Heart function is restored through growth factors such as FGF2 and VEGFA, many types of miRNAs, exosomes that can regulate various physiological functions of myocardial cells by promoting vascular regeneration, myocardial cell proliferation, or inhibiting fibrosis, myocardial necrosis, and ROS levels. The other type of therapy functions in the cellular level and mainly uses cells with differentiation potential, such as MSCs, heart-derived cells and iPSCs, and induces these cells to differentiate into CMs or reprograms fibroblasts to differentiate into iCMs or cardiac progenitor cells.

Immune cells in the heart are mainly divided into resident and recruited cells. Resident immune cells, cTMs, NK cells and recruited immune cells play a role in the heart. Through cell-cell interactions, these cells phagocytose bacteria and necrotic cells and regulate proliferation, inflammation, fibrosis, and extracellular matrix and collagen formation to maintain normal heart functions.

The immune cell repair response to cardiac injury mainly manifests as three overlapping phases: inflammation, proliferation and maturation. In the inflammatory phase, inflammatory cells are recruited, proinflammatory factors are produced, and necrotic cardiomyocytes and cell debris are engulfed or cleared. In the proliferation phase, there is proliferation of cardiomyocytes and fibroblasts, angiogenesis, fibrosis and extracellular matrix formation. In the maturation stage, the recruitment of inflammatory cells is suppressed, anti-inflammatory cytokines are produced, and ultimately inflammation and scar formation are eliminated. Maintaining an appropriate balance between the inflammatory phase and the proliferation phase is the key to achieving the best repair outcome. Proper and timely limitation and elimination of inflammation are key elements in the quality of cardiac healing.

Though many therapies including molecular and cell methods have been applied, a lot of work and experimental data have accumulated in the role of the immune microenvironment in the cardiac injury repair, there still are some limitations on the current studies/data in cardiac injury repair. Most therapies of cardiac injury are only used in animals such as mice, zebrafish or animal and human cells, there is an unmet clinical need to treat cardiac injury. Studies mainly focus on cardiac ischemic injury such as IRI and MI, but there are other types of cardiac injury, for example, cardiac injury is caused trauma, heredity or virus infection. In the future, we should strengthen the transformation of current research into the clinical direction, build more different types of injury models, and expand the curable group of patients with cardiac injury t through the combination of basic research and clinical applications.
